# The CIMP Phenotype in *BRAF* Mutant Serrated Polyps from a Prospective Colonoscopy Patient Cohort

**DOI:** 10.1155/2014/374926

**Published:** 2014-04-10

**Authors:** Winnie C. Fernando, Mariska S. Miranda, Daniel L. Worthley, Kazutomo Togashi, Dianne J. Watters, Barbara A. Leggett, Kevin J. Spring

**Affiliations:** ^1^Conjoint Gastroenterology Laboratory, QIMR Berghofer Medical Research Institute, 300 Herston Road, Brisbane, QLD 4029, Australia; ^2^School of Biomolecular and Physical Sciences, Griffith University, Brisbane, QLD 4111, Australia; ^3^The School of Medicine, The University of Queensland, Brisbane, QLD 4006, Australia; ^4^Department of Coloproctology, Aizu Medical Center, Fukushima Medical University, Fukushima 960-1295, Japan; ^5^School of Biomolecular and Physical Sciences and Eskitis Institute for Drug Discovery, Griffith University, Brisbane, QLD 4111, Australia; ^6^Department of Gastroenterology and Hepatology, The Royal Brisbane and Women's Hospital, Brisbane, QLD 4029, Australia; ^7^Ingham Institute, Liverpool Hospital and Liverpool Clinical School, University of Western Sydney, Sydney, NSW 2170, Australia

## Abstract

Colorectal cancers arising via the serrated pathway are often associated with *BRAF* V600E mutation, CpG island methylator phenotype (CIMP), and microsatellite instability. Previous studies have shown a strong association between *BRAF* V600E mutation and serrated polyps. This study aims to evaluate CIMP status of all the serrated polyp subtypes and its association with functionally important genes such as *MLH1, p16,* and *IGFBP7*. CIMP status and methylation were evaluated using the real-time based MethyLight assay in 154 serrated polyps and 63 conventional adenomas. Results showed that CIMP-high serrated polyps were strongly associated with *BRAF* mutation and proximal colon. CIMP-high was uncommon in conventional adenomas (1.59%), occurred in 8.25% of hyperplastic polyps (HPs), and became common in sessile serrated adenomas (SSAs) (51.43%). *MLH1* methylation was mainly observed in the proximal colon and was significantly associated with *BRAF* mutation and CIMP-high. The number of samples methylated for *p16* and *IGFBP7* was the highest in SSAs. The methylation panel we used to detect CIMP is highly specific for CIMP-high cancers. With this panel, we demonstrate that CIMP-high is much more common in SSAs than HPs. This suggests that CIMP-high correlates with increased risk of malignant transformation which was also observed in methylation of functionally important genes.

## 1. Introduction

Colorectal cancer (CRC) is a commonly occurring cancer with significant mortality in many countries. It is also a heterogeneous disease, which arises from two main groups of precursor lesions or polyps-conventional adenomas (CAs) and serrated polyps. Conventional adenomas are classified into tubular adenoma (TA), tubulovillous adenoma (TVA), and villous adenoma (VA) [1, 2]. Conventional adenomas give rise to CRC via the traditional pathway which is characterised by inactivation of the* adenomatous polyposis coli (APC) *gene, multiple allelic losses on chromosomes 5q, 17p, and 18q, inactivation of tumour suppressor genes such as* p53*,and epigenetic DNA hypomethylation [3, 4].

Serrated polyps were initially considered as lesions with no malignant potential; however, studies identified an alternate route to colorectal tumorigenesis arising from serrated polyps via the serrated neoplastic pathway [5–8]. Serrated polyps are classified into hyperplastic polyps (HPs) comprising microvesicular HP (MVHP), goblet cell HP (GCHP), and mucin poor HP (MPHP), traditional serrated adenoma (TSA), and sessile serrated adenoma (SSA)-with cytological dysplasia and without cytological dysplasia [9, 10]. The colorectal tumours arising from serrated polyps have* BRAF *mutations, CpG island methylation, and often high levels of microsatellite instability (MSI-H) [11–13].

Hypermethylation is one of the key features of the serrated pathway and causes inactivation of multiple functionally important genes such as* MLH1*,* p16*,* MGMT*,* TIMP3*, and* HLTF *[13, 14]. Toyota et al. classified methylation patterns in CRC into two categories-type A or age-specific methylation and type C or cancer-specific methylation. In this study, a group of colorectal tumours exhibited high type C methylation levels in 3 or more loci and this feature was described as CpG island methylator phenotype (CIMP). CIMP is an event in colorectal tumorigenesis and has the potential to silence key tumour suppressor genes [13]. CIMP was found to be strongly associated with* BRAF* mutation, MSI-H, and proximally located colorectal tumours [15, 16].

In 2006, Weisenberger et al. investigated CIMP in greater detail and developed a new panel of methylation markers to more specifically classify colorectal tumours based on their CIMP status. The MethyLight assay, a real-time based PCR technique, was employed to assess the methylation levels in the tumours. This study developed a panel of markers which more accurately defined tumours with high levels of CIMP (CIMP-H) and which were closely associated with* BRAF *mutation [17, 18]. In polyps, CIMP assessment using nonspecific markers and nonquantitative methylation-specific PCR (MSP) showed that CIMP was frequent in all serrated polyps, except for GCHPs [8]. It is likely that CIMP develops in serrated polyps and plays a role in their progression to malignancy. Although the Weisenberger et al. panel is more specific and quantitative, it has not been used previously to assess CIMP in all the serrated polyp types [8, 17, 19].

The aim of our study was to evaluate CIMP status using the Weisenberger et al. panel and MethyLight assay in a well-characterised series of serrated polyps, which were used in an earlier study [20]. Our hypotheses were that (a) CIMP will be rare in HPs and CAs but will be more prevalent in SSAs which are the immediate precursors to CIMP-H and MSI-H CRC and (b) CIMP will be more prevalent in proximal lesions and (c) will be associated with the methylation of genes whose silencing is thought to play a functional role in the serrated neoplastic pathway (*MLH1*,* p16*, and* IGFBP7*) [21].

## 2. Materials and Methods

### 2.1. Patients and Colorectal Polyp Samples

A cohort of 154 serrated polyps and 63 conventional adenomas were used in this study. All of these polyps were obtained from 112 patients derived from a larger consecutive unselected series of 189 patients who underwent colonoscopy at the Royal Brisbane and Women's Hospital (RBWH) in 2003 as previously described by Spring et al. [20]. All the patients gave written informed consent, and the Human Research Ethics Committee at the RBWH approved the study. Patients with familial adenomatous polyposis (FAP), hereditary nonpolyposis CRC (HNPCC) and hyperplastic polyposis (HPP) were excluded from this study. Polyp location, histology, gender, and size were recorded at the time of collection. The 217 polyps included in this study consisted of 35 sessile serrated adenomas (SSAs), 3 traditional serrated adenomas (TSAs), 7 mixed polyps (MPs), 59 goblet cell (GC) hyperplastic polyps (HPs), 50 microvesicular (MV) hyperplastic polyps (HPs), 11 tubulovillous adenomas (TVAs), and 52 tubular adenomas (TAs). Mixed polyps consisted of 3 HPs with CA (2 GCHP and 1 MVHP), 3 HPs mixed with TSA (2 MVHP and 1 GCHP), and 1 TVA mixed with TSA. The 154 serrated polyps identified from the original series were from 75 patients, whilst the remaining patients (114) had conventional adenomas only. Of the 154 serrated polyps in this study, a further seven GCHPs, four MVHPs, and one SSA were excluded due to failure of these samples to amplify in the MethyLight assay.

### 2.2. Methylation Analysis

DNA was extracted from formalin-fixed paraffin-embedded (FFPE) tissues using the Chelex method as described in the Spring et al. study [20]. 500 ng of DNA was subjected to bisulfite modification using EpiTect Bisulfite kit (Qiagen, Limburg, The Netherlands), and the MethyLight assay was used to analyse the methylation levels in bisulfite modified samples [18]. CIMP status was assessed using the Weisenberger et al. panel of CIMP markers-*IGF2*,* SOCS1*,* NEUROG1*,* RUNX3*, and* CACNA1G* (Table 1) [17]. The methylation of* MLH1*,* p16*,and* IGFBP7 *was also analysed using the MethyLight assay using the following cycling conditions-*ALU*,* IGFBP7*,* MLH1*,* NEUROG1*, and* CACNA1G*: 15 sec at 95°C and 60 sec at 60°C;* IGF2*,* SOCS1*,and* p16*: 10 sec at 95°C and 30 sec at 60°C; and* RUNX3*: 15 sec at 95°C and 60 sec at 62°C. The level of methylation was calculated based on the percentage of methylated reference (PMR) of each sample with* ALU* as the standard, and samples with a PMR ≥7 were considered to be methylated. Polyps were designated as CIMP-H if three or more of the panel markers were methylated. The methylation and CIMP status of polyps were correlated with* BRAF* V600E and* KRAS *mutations, polyp location, size, histology, and patient gender.

### 2.3. Screening for* BRAF* V600E and* KRAS *Mutations

The polyps were screened for* BRAF* V600E and* KRAS* mutations (codons 12 and 13) using the allele-specific PCR and the MassARRAY system as previously described [20].

### 2.4. Statistical Analysis

*GraphPad Prism 6 V 6.02* software was used to perform the statistical analysis. Fisher's exact test and Chi-square test were used to calculate *P* values (two-tailed) depending on the data analyzed.

## 3. Results

### 3.1. CIMP and Polyp Type

**Figure 1:**
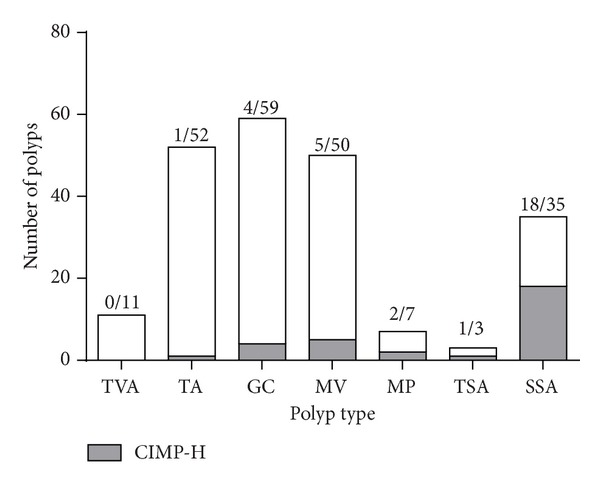
*CIMP-H in different polyp types*. TAs and TVAs comprise the conventional adenoma control group, while the serrated polyps consist of GCs, MVs (hyperplastic polyps) and MPs, TSAs, and SSAs. CIMP-H status was found to be significantly associated with serrated polyps (*P* = 0.0002). SSAs had the highest number of CIMP-H polyps followed by HPs.

Serrated polyps were found to be significantly associated with CIMP-H status (Figure 1 and Table 2). The SSAs showed the highest percentage of CIMP-H samples (51.43%), while only nine out of 109 HPs (8.26%) were CIMP-H. The number of CIMP-H HPs was similar in GCHPs and MVHPs with 6.78% and 10%, respectively. In TVAs, CIMP-H samples were absent and they accounted for only a minor portion in TAs, one out of 52 TAs (1.92%).

### 3.2. CIMP and Anatomic Location, Polyp Size, and Gender

**Figure 2:**
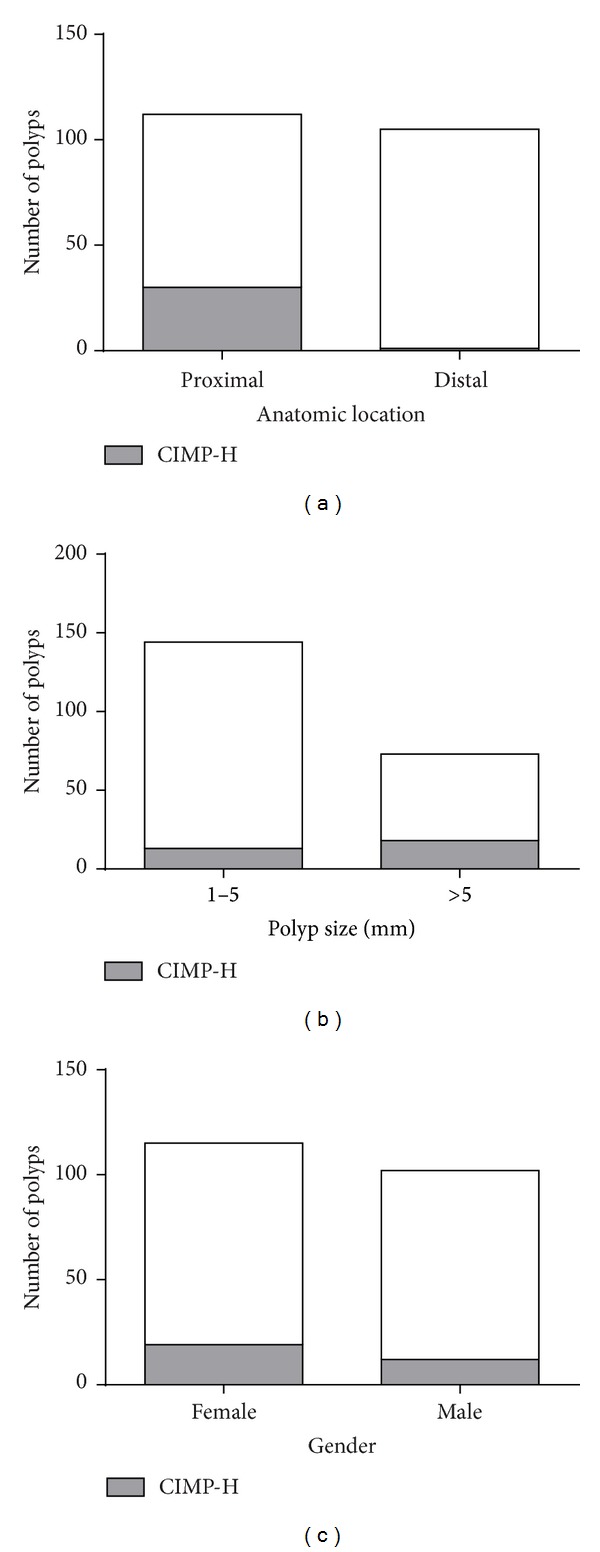
*Correlation between CIMP and location, polyp size, and gender*. CIMP-H polyps displayed a significant correlation with proximal location (a) and large polyp size (>5 mm) (b). However, there was no significant correlation between CIMP-H and gender, although females had a higher number of CIMP-H polyps than males (c).

CIMP-H was significantly associated with proximal colonic location-30 out of 112 (26.79%) polyps in the proximal colon were CIMP-H as shown in Figure 2(a) (*P* < 0.0001). A majority of the SSAs were proximally located-27 out of 35 (77.14%); 17 of 27 proximal SSAs were CIMP-H (62.96%). The remaining eight SSAs were located in the distal colon and only one was CIMP-H (12.5%). In the HPs, a similar pattern was observed-all the CIMP-H samples (four GCHPs and five MVHPs) were located in the proximal colon.

Overall, the size of the polyp also had an effect on the CIMP status. CIMP-H increased significantly (*P* = 0.0035) with an increase in polyp size as shown in Figure 2(b). However, this was not the case for SSAs, which were just as likely to be CIMP-H even if they were small. In the size range of 1–5 mm, six of 12 SSAs (50%) were CIMP-H. In SSAs > 5 mm, 12 of 23 (52.17%) were CIMP-H.

There was no significant association between CIMP-H polyps and gender. Females showed a slightly higher incidence of CIMP-H polyps as seen in Figure 2(c) with 19 CIMP-H samples out of 115 (16.52%), while males had 12 CIMP-H polyps out of a total of 102 (11.76%).

### 3.3. *BRAF *Mutant/CIMP-H Status and Polyp Type with respect to Location

As reported in Spring et al,* BRAF* mutation was strongly associated with MVHPs and SSAs and* KRAS* mutation was associated with GCHPs and TVAs [20]. The only TA with a* BRAF* mutation was a <5 mm polyp occurring in the setting of multiple SSAs and was the only TA to show CIMP-H. There were no TVAs with* BRAF* mutation or CIMP-H status (Table 2).

*BRAF *mutations were common in MVHPs-36* BRAF* mutant polyps out of 50 and three were* BRAF* mutant/CIMP-H. The other two CIMP-H MVHPs were wild-type for both* BRAF *and* KRAS*. Although* KRAS* mutation was predominant in GCHPs and only 20.34% had* BRAF* mutations, three out of the four CIMP-H samples were* BRAF* mutant and the remaining CIMP-H sample was wild-type for both* BRAF *and* KRAS*. TSAs and MPs had the same proportion of* BRAF *mutant/CIMP-H polyps (50%). SSAs had a high percentage of* BRAF *mutant polyps (80%) and had the highest number of* BRAF *mutant/CIMP-H polyps - 18 out of a total of 28* BRAF *mutant samples (64.29%) (see Table S1 in the Supplementary Material available online at http://dx.doi.org/10.1155/2014/374926). Overall, there was a very significant association between* BRAF* mutation and CIMP-H (*P* < 0.0001) and a negative association between* KRAS* mutation and CIMP-H (Supplementary Table S2).

**Figure 3:**
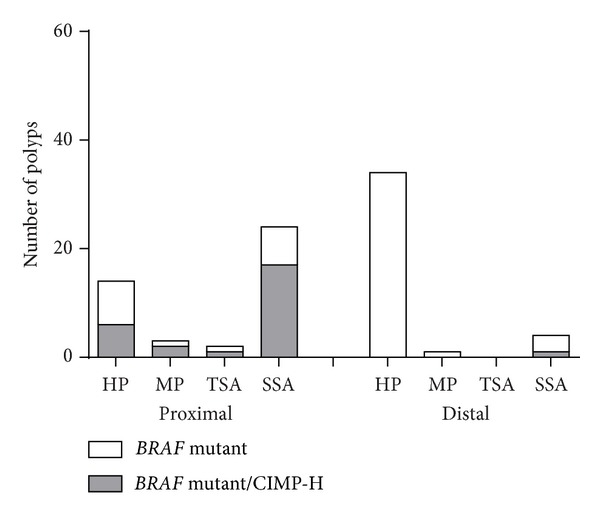
*BRAF* mutation and* CIMP in serrated polyps*. A comparative analysis of the distribution of* BRAF* mutant/CIMP-H samples in serrated polyps with respect to the anatomical location in the human colon.

The distribution of* BRAF *mutant/CIMP-H in serrated polyps was assessed with respect to anatomic location (Figure 3). In the proximal region, SSAs had a significant (*P* = 0.0335) number of* BRAF* mutated polyps with 24 out of 28 samples (85.71%), out of which 17 SSAs were CIMP-H (70.83%). The SSAs had the least number of distally located* BRAF* mutant samples with only four out of 28 polyps (14.29%) and only one* BRAF* mutant/CIMP-H SSA in the distal colon. In MPs and TSAs, all the* BRAF *mutant and* BRAF *mutant/CIMP-H polyps were proximal, except for one* BRAF *mutant distal MP. Although* BRAF *mutant HPs were more common distally, only proximal HPs showed CIMP-H (Figure 3).

### 3.4. *MLH1* Methylation

*MLH1* methylation was not observed in conventional adenomas and was present in only one MVHP out of 109 HPs (2%). The SSAs had the highest number of* MLH1* methylated samples with seven out of 35 (20%) and it was absent in MPs and TSAs. A significant association between methylation of* MLH1* and CIMP-H status was observed (*P* = 0.0001).* BRAF* V600E mutation was also significantly associated with* MLH1 *methylation (*P* = 0.0056) and was present in seven out of eight* MLH1 *methylated samples. All the polyps with* MLH1* methylation were located in the proximal region except for the one MVHP.

### 3.5. Methylation of* p16* and* IGFBP7*

**Figure 4:**
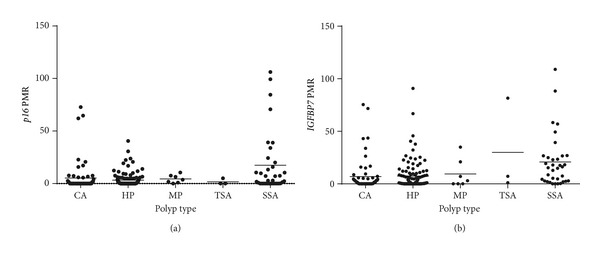
*p16 and IGFBP7 methylation in different polyp types*. The assessment of methylation levels of* p16* (a) and* IGFBP7 *(b) by scatter plots showed that the majority of the* p16* and* IGFBP7* methylated polyps were SSAs followed by HPs. A large number of conventional adenomas also showed low levels of* p16* and* IGFBP7* methylation.

The number of* p16 *methylated samples was the highest in SSAs with 17 of 35 (48.57%) [Figure 4(a)]. In the conventional adenomas only ten samples were methylated for* p16* (15.87%) with the majority being TAs. The levels of* IGFBP7* methylation were similar to the* p16* methylation analysis with the highest number of* IGFBP7* methylated samples in the SSAs with 22 out of 35 samples (62.86%) followed by HPs-35* IGFBP7 *methylated samples out of 109 (32.11%). In conventional adenomas, only 10 out of 63 samples (19.01%) were methylated for* IGFBP7 *[Figure 4(b)].

### 3.6. *p16* and* IGFBP7* Methylation in* BRAF* Mutant and CIMP-H Polyps

**Figure 5:**
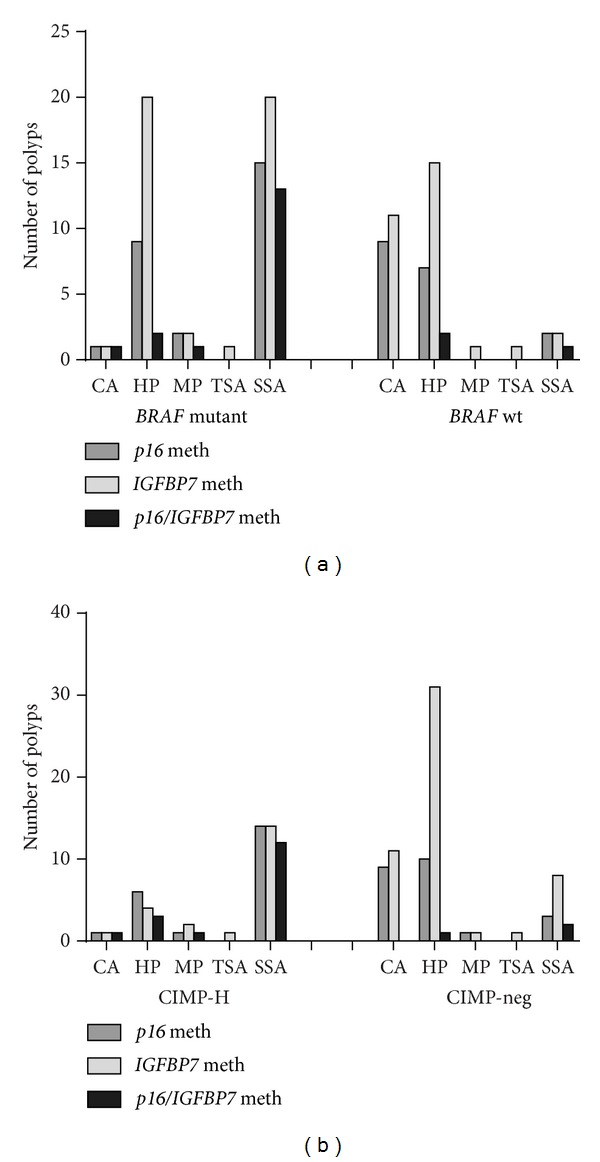
*BRAF mutation and CIMP status in p16 and IGFBP7 methylated polyps*. A comparative analysis was performed to assess the percentage of* BRAF *mutant (a) and CIMP-H (b) samples with* p16* and* IGFBP7 *methylation. The SSAs showed the highest number of* p16* and combined* p16*/*IGFBP7* methylated samples in the* BRAF* mutant and CIMP-H groups and this was closely followed by the HPs.

*BRAF* mutant SSAs had the highest number of* p16* methylated (53.57%),* IGFBP7* methylated (71.43%), and* p16*/*IGFBP7* methylated (46.43%) samples [Figure 5(a)]. In the* BRAF *mutant HPs, 20 out of 48 samples (41.67%) were* IGFBP7 *methylated, but only nine out of 48 samples were methylated for* p16*. The combined* p16*/*IGFBP7* methylation percentage in the HPs was quite low with only two methylated samples compared to the SSAs with combined* p16*/*IGFBP7* methylation in 13 out of 28* BRAF* mutant polyps. The analysis of* p16* and* IGFBP7 *methylation in CIMP-H polyps was shown in Figure 5(b). In CIMP-H SSAs, 77.78% were methylated for* p16 *and* IGFBP7* and combined* p16*/*IGFBP7* methylation was 66.67%. In the CIMP-H HPs, out of nine samples, six and four samples were methylated for* p16* and* IGFBP7*, respectively (Supplementary Table S1).* IGFBP7 *methylation was also common in CIMP-negative HPs.

## 4. Discussion

Toyota et al. identified the existence of CIMP in colorectal tumours and preneoplastic adenomatous polyps [13, 22]. There is also a significant association between CIMP-H, MSI, and* BRAF *mutations in colorectal cancers [23]. In colorectal polyps, CIMP was significantly associated with serrated polyps. CIMP-H conventional adenomas constituted a minor proportion and were dependent on the polyp size (>2 cm) and percentage of villous component (>80%) [24, 25]. A variety of methylation markers have been employed to determine CIMP status in polyps including the Toyota et al. and Weisenberger et al. panels,* HIC1*,* MGMT,* and* RASSF2* using MSP (nonquantitative) and MethyLight (quantitative) assays with a PMR cut-off for methylation ranging from >4 to >10 [24, 26, 27].

Several studies have been performed to classify serrated polyps based on their CIMP status, but a majority of them were retrospective, did not include all serrated polyp types, and were represented by small sample numbers [19, 24, 27–31]. The choice of methylation markers and the assay does affect the outcome of methylation status in samples. The Weisenberger et al. panel was more specific than the Toyota et al. markers for identifying CIMP phenotype associated with* BRAF* mutant and MSI-H colorectal tumours, and quantitative MethyLight assay was more sensitive and specific for methylation analysis compared to nonquantitative MSP [17, 18].

Our study is one of the first prospective studies to assess the CIMP status of all the colorectal polyp subtypes using the Weisenberger et al. panel of methylation markers and quantitative MethyLight technology. Our results indicated that CIMP-H status was significantly associated with serrated polyps, proximal location,* BRAF* V600E mutations, and increase in polyp size. This study shows a progressive increase in CIMP as the malignant potential of serrated polyps increases (HP versus SSA). CIMP seems more likely to develop when there is a* BRAF* mutation and the polyp is in the proximal colonic environment. SSAs had the highest number of CIMP-H polyps (51.43%), while the HPs and CAs showed a very low frequency of CIMP-H with 8.25% and 1.59%, respectively. A majority of other studies showed that SSAs were predominantly CIMP-H, followed by HPs with a higher frequency compared to our study. This could be due to the classification of serrated polyps based on CIMP-H status using MSP and the Toyota et al. methylation markers [8, 19, 32].

In our study, 62.96% of proximal SSAs were CIMP-H and only one CIMP-H SSA was located in the distal colon, while all the CIMP-H HPs were proximal. This was similar to other reports where a majority of CIMP-H SSAs and HPs were proximally located [19, 27, 33]. We found that CIMP-H was significantly associated with* BRAF *mutant serrated polyps and a negative association existed between* KRAS* mutation and CIMP-H which was also observed in studies by O'Brien et al. and Yang et al. [19, 29]. Based on our results and similar studies,* BRAF* mutant, CIMP-H SSAs which are proximally located provide further support for SSAs as potential precursors of* BRAF* mutant, CIMP-H CRCs. A recent retrospective CIMP study by Burnett-Hartman et al. was carried out on 359 serrated polyps using the Weisenberger et al. methylation panel and MethyLight assay. Results showed that CIMP-H was more prevalent in SSAs, proximal colon, and* BRAF* mutant serrated lesions, which confirms our findings [31].

*MLH1* and* p16* along with* MINT1*,* MINT2*, and* MINT31* constitute the Toyota et al. panel [13].* p16* and* MLH1* methylation have been evaluated in numerous studies in colorectal tumours and polyps. In tumours,* MLH1* methylation showed a significant association with CIMP, MSI-H and was mainly localized to the proximal colon [13, 34]. In HPs, 3–21% of the samples were methylated for* MLH1*, while the frequency varied from 16 to 23.2% in SSAs [27, 32, 35, 36]. Only 7% of the TAs were methylated for* MLH1*, while this was completely absent in the TVAs [25, 35]. Out of 217 polyps in our study, only 3.69% of the samples were methylated for* MLH1* and all the samples were proximally located except for one MVHP.* MLH1* methylation was detected in 4% of HPs and 17% of SSAs and was completely absent in conventional adenomas and these results were found to be similar to reports discussed above [27, 31].

*p16 *methylation is also closely linked to CIMP-H and MSI-H CRCs [13, 37, 38]. The results from our study indicated that* p16* methylation was correlated with serrated polyp type, proximal location,* BRAF* V600E mutations, and CIMP-H status. In a study by Dhir et al.,* p16* along with* MLH1*,* CDX2, *and* TLR2 *showed the highest frequency of methylation in SSAs based on an unsupervised clustering analysis [39]. A similar trend was observed in other studies with the frequency of* p16* methylation being the highest in SSAs with 76.8%, while it was 48% in TSAs and 29% in TAs [24, 32]. Our results showed a comparatively lower percentage of* p16* methylated samples in SSAs (48.57%) and TAs (11.53%). There was no increase in* p16* methylation from TAs to TVAs as was observed by Psofaki et al. [38].

*IGFBP7* has been implicated as an early event in CRC progression via the serrated pathway. Methylation of* IGFBP7*, a potential tumour suppressor gene, was also evaluated in different polyp types. Similar to* p16* methylation,* IGFBP7* methylated polyps were mainly serrated, closely linked to* BRAF* V600E mutations and CIMP-H status. A study by Suzuki et al. also observed similar correlations between* IGFBP7 *methylation and* BRAF* mutation and* p16* methylation and CIMP and an inverse correlation with* KRAS* mutation in colorectal tumours.* IGFBP7* methylation was also detected in 18% of conventional adenomas, which was similar to our results (19%) [40]. In a recent study by Kaji et al, it was proposed that* IGFBP7* methylation did not affect HPs due to lower PMRs when compared to TSAs or SSAs [41]. This was also observed in our study with only 32.11% of HPs methylated for* IGFBP7* which was lower than TSAs (66.67%) and SSAs (62.86%) [40, 41].

## 5. Conclusion

To date, the serrated pathway has not yet been fully characterised. Our study showed that CIMP-H was significantly associated with serrated polyps, mainly the SSA subgroup. Only a minority of HPs and CAs were CIMP-H. All the CIMP-H HPs and a significant proportion of CIMP-H SSAs were located in the proximal colon. There was a strong association between* BRAF* V600E mutation and CIMP-H polyps. Methylation of the functionally important genes* MLH1*,* p16*, and* IGFBP7* was the highest in SSAs and was associated with* BRAF* V600E mutation, CIMP-H, and proximal location. Our study using a stringent definition of CIMP has highlighted the association of increasing levels of methylation in serrated polyps as their malignant potential increases. The application of advanced methodology such as methylation and expression arrays will help to evaluate more genes/markers and hopefully contribute towards a more defined understanding of the various changes causing the transformation of a serrated precursor lesion into CRC.

## Supplementary Material

The Supplementary material includes comparative analyses between the different molecular features such as *BRAF* V600E and *KRAS* mutations, CIMP-H and methylation of *MLH1*, *p16* and *IGFBP7* and the polyp types. Supplementary Table S1 entitled 'Serrated polyp subtypes and their association with *BRAF* V600E and *KRAS* mutations, CIMP-H status and *MLH1*, *p16* and *IGFBP7* methylation' shows the prevalence of important molecular features in all the serrated polyp types. *BRAF* and *KRAS* mutant and CIMP-H serrated polyps were correlated with methylation of *MLH1*, *p16* and *IGFBP7*. Supplementary Table S2 entitled ‘Association between CIMP status, *BRAF* V600E and *KRAS* mutations in all polyp types' is a comparative analysis between CIMP-H and CIMP-negative cases and *BRAF* and *KRAS* mutational status in all polyp types.

## Figures and Tables

**Table 1:** Primers and probes for methylation, *BRAF*, and *KRAS* mutation analysis.

Gene	Primers
*ALU *	**FP**: GGTTAGGTATAGTGGTTTATATTTGTAATTT
	**RP**: ATTAACTAAACTAATCTTAAACTCCTAACCT
	**Probe**: 6FAM-CCTACCTTAACCTCCC-MGBNFQ

*IGF2 *	**FP**: GAGCGGTTTCGGTGTCGTTA
	**RP**: CCAACTCGATTTAAACCGACG
	**Probe**: 6FAM-CCCTCTACCGTCGCGAACCCGA-BHQ-1

*SOCS1 *	**FP**: GCGTCGAGTTCGTGGGTATTT
	**RP**: CCGAAACCATCTTCACGCTAA
	**Probe**: 6FAM-ACAATTCCGCTAACGACTATCGCGCA-BHQ-1

*NEUROG1 *	**FP**: CGTGTAGCGTTCGGGTATTTGTA
	**RP**: CGATAATTACGAACACACTCCGAAT
	**Probe**: 6FAM-CGATAACGACCTCCCGCGAACATAAA-BHQ-1

*RUNX3 *	**FP**: CGTTCGATGGTGGACGTGT
	**RP**: GACGAACAACGTCTTATTACAACGC
	**Probe**: 6FAM-CGCACGAACTCGCCTACGTAATCCG-BHQ-1

*CACNA1G *	**FP**: TTTTTTCGTTTCGCGTTTAGGT
	**RP**: CTCGAAACGACTTCGCCG
	**Probe**: 6FAM-AAATAACGCCGAATCCGACAACCGA-BHQ-1

*MLH1 *	**FP**: AGGAAGAGCGGATAGCGATTT
	**RP**: TCTTCGTCCCTCCCTAAAACG
	**Probe**: 6FAM-CCCGCTACCTAAAAAAATATACGCTTACGCG-BHQ-1

*p16 *	**FP**: TGGAGTTTTCGGTTGATTGGTT
	**RP**: AACAACGCCCGCACCTCCT
	**Probe**: 6FAM-ACCCGACCCCGAACCGCG-BHQ-1

*IGFBP7 *	**FP**: GGTAAAGTCGGGGTAGTAGTCG
	**RP**: ACAACCGCTCGAATAAATAATACCG
	**Probe**: 6FAM-CGCTACCGCACACCGAATAACGACTCTTA-BHQ-1

*BRAF *	**V**: GTGATTTTGGTCTAGCTACtG***T***
	**E**: CGCGGCCGGCCGCGGCGGTGATTTTGGTCTAGCTACcGA
	**AS**: TAGCCTCAATTCTTACCATCCAC

*KRAS *189-bp	**FP**: TCATTATTTTTATTATAAGGCCTGCTGAA
	**RP**: CAAAGACTGGTCCTGCACCAGTA

*KRAS *92-bp	**FP**: ttataagGCCTGCTGAAAATGACTGAA
	**RP**: TGAATTAGCTGTATCGTCAAGGCACT

**Table 2:** Correlation between polyp type, location, gender, size, *BRAF* V600E and *KRAS* mutations, CIMP, and methylation of *MLH1*, *p16,* and *IGFBP7*.

Clinical, pathological, and molecular features	All cases	*BRAF* mutant	*KRAS* mutant	CIMP-H	*MLH1 *methylation	*p16 *methylation	*IGFBP7 *methylation	*IGFBP7* and *p16 *methylation
All colorectal polyps	217	83 (38.25%)	51 (23.5%)	31 (14.29%)	8 (3.69%)	45 (20.74%)	74 (34.1%)	20 (9.22%)
Patient age (years, SD)	62.36 ± 13.54	58.8 ± 13.48	61.67 ± 13.87	64 ± 13.51	71 ± 6.05	63.4 ± 14.17	58.45 ± 13.3	58.8 ± 14.98
Gender								
Female	115	50 (43.48%)	26 (22.61%)	19 (16.52%)	4 (3.48%)	28 (24.35%)	49 (42.61%)	16 (13.91%)
Male	102	33 (32.35%)	25 (24.51%)	12 (11.76%)	4 (3.92%)	17 (16.67%)	25 (24.51%)	4 (3.92%)
Location								
Proximal	112	44 (39.29%)	18 (16.07%)	30 (26.79%)	7 (6.25%)	38 (33.93%)	43 (38.39%)	18 (16.07%)
Distal	105	39 (37.14%)	33 (31.43%)	1 (0.95%)	1 (0.95%)	7 (6.67%)	31 (29.52%)	2 (1.9%)
Polyp type								
Serrated polyps	154	82 (53.25%)	40 (25.97%)	30 (19.48%)	8 (5.19%)	35 (22.73%)	62 (40.26%)	19 (12.34%)
HP	109	48 (44.04%)	35 (32.11%)	9 (8.26%)	1 (0.92%)	16 (14.68%)	35 (32.11%)	4 (3.67%)
GCHP	59	12 (20.34%)	30 (50.85%)	4 (6.78%)	0	7 (11.86%)	19 (32.2%)	2 (3.39%)
MVHP	50	36 (72%)	5 (10%)	5 (10%)	1 (2%)	9 (18%)	16 (32%)	2 (4%)
TSA	3	2 (66.67%)	0	1 (33.33%)	0	0	2 (66.67%)	0
MP	7	4 (57.14%)	2 (28.57%)	2 (28.57%)	0	2 (28.57%)	3 (42.86%)	1 (14.29%)
SSA	35	28 (80%)	3 (8.57%)	18 (51.43%)	7 (20%)	17 (48.57%)	22 (62.86%)	14 (40%)
Conventional adenomas	63	1 (1.59%)	11 (17.46%)	1 (1.59%)	0	10 (15.87%)	12 (19.05%)	1 (1.59%)
TA	52	1 (1.92%)	4 (7.69%)	1 (1.92%)	0	6 (11.54%)	10 (19.23%)	1 (1.92%)
TVA	11	0	7 (63.64%)	0	0	4 (36.36%)	2 (18.18%)	0
Size								
1–5 mm	144	50 (34.72%)	37 (25.69%)	13 (9.03%)	3 (2.08%)	22 (15.28%)	44 (30.56%)	8 (5.56%)
>5 mm	73	33 (45.21%)	14 (19.18%)	18 (24.66%)	5 (6.85%)	23 (31.51%)	30 (41.1%)	12 (16.44%)
*BRAF* status								
*BRAF* mutant	83	—	0	28 (33.73%)	7 (8.43%)	27 (32.53%)	44 (53.01%)	17 (20.48%)
*BRAF* wild	134	—	51 (38.06%)	3 (2.24%)	1 (0.75%)	18 (13.43%)	30 (22.39%)	3 (2.24%)
*KRAS* status								
*KRAS* mutant	51	0	—	0	0	7 (13.73%)	8 (15.69%)	1 (1.96%)
*KRAS *wild	166	83 (50%)	—	31 (18.67%)	8 (4.82%)	38 (22.89%)	66 (39.76%)	19 (11.45%)
