# Supplementary material for: The CIMP Phenotype in BRAF Mutant Serrated Polyps from a Prospective Colonoscopy Patient Cohort
Source: Gastroenterol Res Pract. 2014 Apr 10;2014:374926. doi: 10.1155/2014/374926 (PMC4000649; doi:10.1155/2014/374926)
Supplement: Supplementary file 1 — The Supplementary material includes comparative analyses between the different molecular features such as BRAF V600E and KRAS mutations, CIMP-H and methylation of MLH1, p16 and IGFBP7 and the polyp types. Supplementary Table S1 entitled 'Serrated polyp subtypes and their association with BRAF V600E and KRAS mutations, CIMP-H status and MLH1, p16 and IGFBP7 methylation' shows the prevalence of important molecular features in all the serrated polyp types. BRAF and KRAS mutant and CIMP-H serrated polyps were correlated with methylation of MLH1, p16 and IGFBP7. Supplementary Table S2 entitled ‘Association between CIMP status, BRAF V600E and KRAS mutations in all polyp types' is a comparative analysis between CIMP-H and CIMP-negative cases and BRAF and KRAS mutational status in all polyp types. [file 374926.f1.pdf]

Supplementary Table S1: ***Serrated polyp subtypes and their association with BRAF V600E and KRAS mutations, CIMP-H status and MLHI, p16 and IGFBP7 methylation***

**Clinical, pathological & molecular features**	**All cases**	***BRAF* mutant**	***KRAS* mutant**	**CIMP-H**	***MLH1* methylation**	***p16* methylation**	***IGFBP7* methylation**	***IGFBP7* & *p16* methylation**
**Serrated polyps**	154	82 (53.25%)	40 (25.97%)	30 (19.48%)	8 (5.19%)	35 (22.73%)	62 (40.26%)	19 (12.34%)
BRAF mutant								
**Serrated polyps**	**82**	**-**	**0**	**27 (32.93%)**	**7 (8.54%)**	**26 (31.71%)**	**43 (52.44%)**	**16 (19.51%)**
HP	48	-	0	6 (12.5%)	1 (2.08%)	9 (18.75%)	20 (41.67%)	2 (4.17%)
GCHP	*12*	-	*0*	*3 (25%)*	*0*	*3 (25%)*	*7 (58.33%)*	*1 (8.33%)*
MVHP	*36*	-	*0*	*3 (8.33%)*	*1 (2.78%)*	*6 (16.67%)*	*13 (36.11%)*	*1 (2.78%)*
TSA	2	-	0	1 (50%)	0	0	1 (50%)	0
MP	4	-	0	2 (50%)	0	2 (50%)	2 (50%)	1 (25%)
SSA	28	-	0	18 (64.29%)	6 (21.43%)	15 (53.57%)	20 (71.43%)	13 (46.43%)
KRAS mutant								
**Serrated polyps**	**40**	**0**	**-**	**0**	**0**	**3 (7.5%)**	**6 (15%)**	**1 (2.5%)**
HP	35	0	-	0	0	3 (8.57%)	5 (14.29%)	1 (2.86%)
GCHP	*30*	*0*	-	*0*	*0*	*2 (6.67%)*	*5 (16.67%)*	*1 (3.33%)*
MVHP	*5*	*0*	-	*0*	*0*	*1 (20%)*	*0*	*0*
TSA	0	0	-	0	0	0	0	0
MP	2	0	-	0	0	0	1 (50%)	0
SSA	3	0	-	0	0	0	0	0
CIMP-H								
**Serrated polyps**	**30**	**27 (90%)**	**0**	**-**	**6 (20%)**	**21 (70%)**	**21 (70%)**	**16 (53.33%)**
HP	9	6 (66.67%)	0	-	0	6 (66.67%)	4 (44.44%)	3 (33.33%)
GCHP	*4*	*3 (75%)*	*0*	-	*0*	*3 (75%)*	*2 (50%)*	*1 (25%)*
MVHP	*5*	*3 (60%)*	*0*	-	*0*	*3 (60%)*	*2 (40%)*	*2 (40%)*
TSA	1	1 (100%)	0	-	0	0	1 (100%)	0
MP	2	2 (100%)	0	-	0	1 (50%)	2 (100%)	1 (50%)
SSA	18	18 (100%)	0	-	6 (33.33%)	14 (77.78%)	14 (77.78%)	12 (66.67%)

Supplementary Table S2: ***Association between CIMP status, BRAF V600E and KRAS mutations in all polyp types***

**Polyp type**	**All cases**	**CIMP-H cases**	**CIMP-H**						**CIMP-negative cases**	**CIMP-negative**					
			KRAS		***BRAF***		***KRAS/BRAF***			***KRAS***		***BRAF***		***KRAS/BRAF***	
			**Mut**	**Wild**	**Mut**	**Wild**	**Mut**	**Wild**		**Mut**	**Wild**	**Mut**	**Wild**	**Mut**	**Wild**
**SP**	**154**	**30 (19.48%)**	**0**	**30 (100%)**	**27 (90%)**	**3 (10%)**	**0**	**3 (10%)**	**124 (80.52%)**	**40 (32.26%)**	**84 (67.74%)**	**55 (44.35%)**	**69 (55.65%)**	**0**	**29 (23.39%)**
HP	109	9 (8.26%)	0	9 (100%)	6 (66.67%)	3 (33.33%)	0	3 (33.33%)	100 (91.74%)	35 (35%)	65 (65%)	42 (42%)	58 (58%)	0	23 (23%)
GCHP	*59*	*4 (6.78%)*	*0*	*4 (100%)*	*3 (75%)*	*1 (25%)*	*0*	*1 (25%)*	*55 (93.22%)*	*30 (54.55%)*	*25 (45.45%)*	*9 (16.36%)*	*46 (83.64%)*	*0*	*16 (29.09%)*
MVHP	*50*	*5 (10%)*	*0*	*5 (100%)*	*3 (60%)*	*2 (40%)*	*0*	*2 (40%)*	*45 (90%)*	*5 (11.11%)*	*40 (88.89%)*	*33 (73.33%)*	*12 (26.67%)*	*0*	*7 (15.56%)*
TSA	3	1 (33.33%)	0	1 (100%)	1 (100%)	0	0	0	2 (66.67%)	0	2 (100%)	1 (50%)	1 (50%)	0	1 (50%)
MP	7	2 (28.57%)	0	2 (100%)	2 (100%)	0	0	0	5 (71.43%)	2 (40%)	3 (60%)	2 (40%)	3 (60%)	0	1 (20%)
SSA	35	18 (51.43%)	0	18 (100%)	18 (100%)	0	0	0	17 (48.57%)	3 (17.65%)	14 (82.35%)	10 (58.82%)	7 (41.18%)	0	4 (23.53%)
**CA**	**63**	**1 (1.59%)**	**0**	**1 (100%)**	**1 (100%)**	**0**	**0**	**0**	**62 (98.41%)**	**11 (17.74%)**	**51 (82.26%)**	**0**	**62 (100%)**	**0**	**51 (82.26%)**
TA	52	1 (1.92%)	0	1 (100%)	1 (100%)	0	0	0	51 (98.08%)	4 (7.84%)	47 (92.16%)	0	51 (100%)	0	47 (92.16%)
TVA	11	0	0	0	0	0	0	0	11 (100%)	7 (63.64%)	4 (36.36%)	0	11 (100%)	0	4 (36.36%)
